# Effects of individual micronutrients on blood pressure in patients with type 2 diabetes: a systematic review and meta-analysis of randomized clinical trials

**DOI:** 10.1038/srep40751

**Published:** 2017-01-13

**Authors:** Tatiana P. de Paula, Caroline K. Kramer, Luciana V. Viana, Mirela J. Azevedo

**Affiliations:** 1 Endocrinology Division, Hospital de Clínicas de Porto Alegre, Universidade Federal do Rio Grande do Sul, Porto Alegre, Brazil; 2 Division of Endocrinology, University of Toronto, Toronto, ON, Canada

## Abstract

To investigate the effects of micronutrients on blood pressure (BP) in patients with type 2 diabetes through a systematic review and meta-analysis, randomized clinical trials (RCTs) of the effects of individual micronutrients on BP in patients with type 2 diabetes were searched in the Medline, Embase, Cochrane, and Clinical Trials.gov databases through April 2016. From the 28,164 studies, 11 RCTs (13 interventions, 723 patients, 54% males) with 3 to 52 weeks of follow-up were classified according to the type of micronutrient intervention: sodium (n = 1), vitamin C (n = 2), vitamin D (n = 7), and magnesium (n = 1). The available data enabled us to perform meta-analyses of vitamins C and D. Vitamin C reduced diastolic BP [WMD −2.88 mmHg (95%CI −5.31, −0.46; P = 0.020)] but not systolic BP [WMD −3.93 mmHg (95%CI −14.78, 6.92; P = 0.478)]. Vitamin D caused a reduction of 4.56 mmHg (WMD; 95%CI −7.65, −1.47; P = 0.004) for systolic BP and 2.44 mm Hg (WMD; 95%CI −3.49, −1.39; P < 0.001) for diastolic BP. In conclusion, vitamin D and possibly vitamin C have beneficial effects on BP in patients with type 2 diabetes. These interventions might represent a novel approach to the treatment of hypertension in these patients.

Hypertension is a major risk factor for chronic complications of diabetes^1^, and approximately 71% of patients with type 2 diabetes in the United States have hypertension^2^. Indeed, most hypertensive type 2 diabetic patients do not achieve optimal blood pressure (BP) levels^3^. Dietary interventions can reduce BP, prevent or delay the development of hypertension, enhance antihypertensive drug efficacy, and decrease cardiovascular risk^4^,^5^, and this evidence highlights the potential therapeutic role of dietary interventions in the management of hypertension.

Dietary recommendations for patients with hypertension include the reduction of sodium intake, moderation of alcohol consumption, and the Dietary Approaches to Stop Hypertension (*DASH*) eating plan^6^,^7^,^8^ recommended by the American Diabetes Association (ADA)^9^. However, dietary advice for hypertension in diabetes^4^,^6^,^9^,^10^,^11^ has been mostly based on studies conducted in non-diabetic subjects^5^,^7^,^8^. In patients with type 2 diabetes, the beneficial association of the DASH diet with BP was previously demonstrated in both a cross-sectional study^12^ and clinical trials^13^,^14^.

The relationship between individual micronutrients and BP in patients with type 2 diabetes mellitus is still uncertain. Data from individual studies^15^,^16^,^17^,^18^,^19^,^20^,^21^,^22^,^23^,^24^,^25^ may not be sufficient to clearly demonstrate the effects of micronutrients on BP. Therefore, the aim of the current study was to evaluate the impact of individual micronutrients on BP in patients with type 2 diabetes through a systematic review and meta-analysis of randomized clinical trials (RCTs).

## Methods

This systematic review was carried out using a protocol designed according to the Cochrane Handbook recommendations^26^ and reported in accordance with the Preferred Reporting Items for Systematic Reviews and Meta-Analyses (PRISMA) statement^27^.

### Data sources and search strategy

We searched the Medline, Embase, Cochrane and ClinicalTrials.gov electronic databases to identify RCTs that reported the effects of micronutrients on BP in patients with type 2 diabetes, with or without hypertension, through April 2016. The initial search strategy is available in the Supplementary Information. The following medical subject headings (MeSH) terms were used: “Blood Pressure” OR “Hypertension” AND “Diabetes Mellitus” AND Micronutrients” OR “Sodium” OR “Magnesium” OR “Ascorbic Acid” OR “Vitamin D”. We also used MeSH terms for macronutrients (carbohydrates, proteins, lipids) to perform a more comprehensive search in addition to the terms related to micronutrients.

### Study selection

Studies were considered eligible for inclusion if they fulfilled all of the following inclusion criteria: presented original data from RCTs assessing the effects of dietary or oral supplementation of a micronutrient on BP values in patients with type 2 diabetes, examined the effect of a micronutrient on BP after a minimum of two weeks of intervention, and reported means (or differences between means) and standard deviations (SD) of BP at baseline and after the intervention. We excluded studies involving children or pregnant women, as well as studies that evaluated more than one micronutrient intervention at the same time or the simultaneous effects of exercise, genetic polymorphisms, and caloric restriction. We also excluded studies that evaluated a specific dietary pattern instead of an individual micronutrient. Crossover trials were excluded if BP was not evaluated before and after each dietary intervention or if there was no description of a washout period between the studied diets.

Hypertension was defined as a systolic BP ≥ 140 mmHg or a diastolic BP ≥ 90 mmHg and/or the use of antihypertensive medication^4^. Studies involving patients who were using antihypertensive medication were not excluded unless the antihypertensive medication was used as an intervention in addition to the supplement.

### Data extraction and quality assessment

Two reviewers (T.P.P. and C.K.K.) independently analysed the titles and abstracts of every paper retrieved by the literature search to identify potentially eligible studies. All articles that did not meet the selection criteria were excluded, and the full text of the remaining papers was obtained for further examination. The reference lists of the retrieved articles were also manually searched (T.P.P. and C.K.K.) for all included studies. The data were extracted independently by the same two investigators with excellent agreement between them (k = 97%). Any disagreements were resolved by a third author (M.J.A. or L.V.V.).

The extracted data (in addition to assessment of office BP changes as a mean (SD), primary or secondary outcome) included the following: type of micronutrient intervention (dietary or supplemental), author’s name, year of publication, trial design, duration of intervention, number of participants, patient characteristics (gender, age, percentage of patients with hypertension, diabetes duration, BMI), and medication use. Information regarding diet characteristics (total energy and macronutrient intake) and evaluation of dietary compliance were extracted from the intervention and control diet descriptions when available.

A quality assessment of our systematic review was performed in order to limit any bias in conducting the study, gain insight into potential comparisons, and guide the interpretation of the findings^26^. To evaluate the quality assessment, two reviewers (T.P. and M.J.A) independently assessed the quality of the methodology of each of the studies included in our systematic review. We used a score based on the Cochrane Collaboration Handbook to assess the individual risk of bias of every included study^26^,^28^. The biases were classified into six categories: selection, performance, detection, attrition, reporting, and other bias^26^,^27^,^28^. We included the assessment of dietary compliance in the “other bias” category. The risk of bias for each individual item was classified as high, low, or unclear. Regarding dietary compliance, the risk of bias was classified as “low” if the study described the method of dietary compliance evaluation.

The quality of the body of evidence in our systematic review was assessed by taking into account the GRADE^26^,^29^ recommendations. The GRADE results provide an optimal primary approach to making decisions regarding imprecision^29^ and allowed us to accurately evaluate the relevance of our systematic review. The GRADE approach includes factors that may decrease (e.g., poor methodological quality, indirectness of evidence, high heterogeneity, imprecision of effect estimates, high risk of publication bias) or increase (e.g., large effect size, absence of spurious effects due to confounding factors, description of the dose-response gradient) the quality of the evidence. Each evaluated factor was rated as high, moderate, low, or very low quality^26^,^28^,^29^. Using this approach, a serious risk of bias was considered if an individual study had more than three unclear or high risk of bias. Imprecision was defined as a meta-analysis confidence interval >0.5. The GRADE quality was evaluated using GRADEpro version 3.6.1 (2004–2001), and all topics of the performed meta-analyses were analysed by the PRISMA checklist^27^.

### Data synthesis and analysis

The descriptive data from the systematic review are presented as mean and/or range whenever possible. Changes in BP for each analysed micronutrient intervention are reported as absolute differences between the arithmetic means at end-of-study and baseline, and weighted mean differences (WMD) were used in the analyses. Changes in the standard deviation (SD) values of BP were directly extracted from the manuscripts or calculated. When SD data were not included, they were calculated, and a correlation of 0.8 between the baseline and final measurements within each group was assumed according to the formula of Follman *et al*.^30^, as proposed by the Cochrane Handbook^30^. We assumed equal variance among the trials and between the intervention and control groups.

Cochrane’s *x* test (Q test) was used to evaluate the heterogeneity between the studies, and a threshold *P* value of 0.1 was considered statistically significant. The *I*^*2*^ test was also performed to evaluate the magnitude of heterogeneity between studies.

The pooled estimate of the mean differences in blood pressure (mmHg) between the intervention and control groups was calculated using a fixed-effect model when there was no heterogeneity or a random-effects model (DerSimonian-Laird method) in the presence of significant heterogeneity (I^2^ > 50%) between the studies.

We performed meta-regression and sensitivity analyses to identify potential sources of heterogeneity in the evaluation of the effects of vitamin D on BP. The covariates used in the meta-regression analyses were chosen by taking into consideration that the variables that seemed to be quite different between the trials, such as different doses of supplements or the age of the patients, could influence the results. Sensitivity analyses were performed to minimize the effects of particular studies that could influence the meta-analysis results. This approach aimed to identify potential outliers by excluding studies such as those with the greatest heterogeneity or the largest sample size.

All statistical analyses were performed using Stata 11.0 software (Stata, College Station, TX, USA). Significance was set at *P* ≤ 0.05, and 95% confidence intervals are included throughout the paper.

## Results

### Literature search

**Figure 1:**
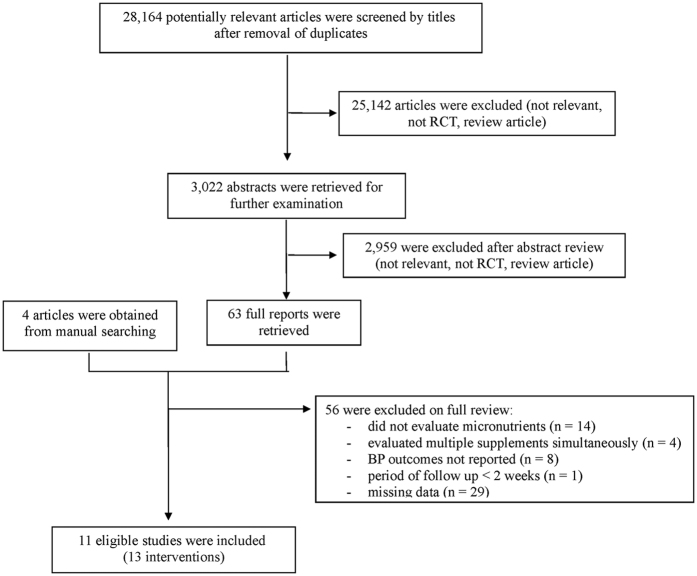
Flow diagram of the literature search to identify randomized clinical trials evaluating the effects of micronutrients on blood pressure in patients with type 2 diabetes.

We identified 28,164 articles in our database searches (Fig. 1), and 28,101 articles were excluded based on the title and abstract. We found four articles by manual searching, leaving 67 manuscripts for full-text evaluation. Among those, 56 articles were excluded due to missing relevant data, a design that was not an RCT, BP outcomes that were not reported, or evaluation of the effects of more than one micronutrient at the same time. Therefore, we selected 11 articles^15^,^16^,^17^,^18^,^19^,^20^,^21^,^22^,^23^,^24^,^25^ that fulfilled all of the inclusion criteria. Two trials reported two interventions each, so 13 interventions from 11 trials were included in the current systematic review.

### Characteristics of the studies

We divided the RCTs into the following four categories according to the type of micronutrient intervention: sodium, vitamin C, vitamin D, and magnesium. The characteristics of the effects of the included micronutrient interventions on BP in patients with type 2 diabetes are summarized in Table 1. Ten reports were parallel RCTs^16^,^17^,^18^,^19^,^20^,^21^,^22^,^23^,^24^,^25^, and one was a crossover-controlled trial^15^. The durations of the trials ranged from 3 to 52 weeks. The included studies provided data from 723 patients with type 2 diabetes and ages ranging from 50.7 to 66.8 years, and 54% of them were males. The duration of diabetes varied from 4.6 to 8.6 years, and six reports^18^,^19^,^20^,^23^,^24^,^25^ did not include this information. The majority of the patients were obese^19^,^20^,^25^ or overweight^16^,^17^,^18^,^23^, and BMI was not reported in four studies^15^,^21^,^22^,^24^. Concerning the descriptions of the main characteristics of the intervention and control diets, the dietary intervention was a supplement in most of the reports (10/11), and the intervention in one study was dietary advice only^15^. The medications in use for diabetes treatment did not differ between the intervention and control groups, and the use of antihypertensive drugs was reported in four studies^15^,^17^,^19^,^24^. The available data from the reviewed RCTs allowed us to perform meta-analyses for vitamin C and vitamin D supplements.

### Summary of Evidence

#### Sodium

Only one study^15^ including 34 hypertensive type 2 diabetic patients with a mean age of 61.4 years (67.6% males) evaluated the effects of sodium on BP. In the first phase of that study, 34 patients were randomly advised to reduce their daily sodium intake for 3 months. A significant reduction of approximately 20 mmHg in supine systolic BP was observed, which was associated with a reduction in daily salt intake of approximately 3 g (corresponding to a daily salt consumption of 11.6 to 8.2 g). The patients who were part of the salt intake reduction arm were included in a one-month crossover-randomized trial to evaluate the effects of sodium supplementation. It was unclear if there was a washout period between the 1^st^ and 2^nd^ phases, and only nine patients completed this study.

#### Magnesium

We identified only one RCT^16^ that examined the effects of magnesium supplementation on BP over three weeks in 82 type 2 diabetic patients with hypertension and hypomagnesaemia (mean 0.62 mmol/l; reference range 0.70 to 0.85 mmol/l). In this double-blind, placebo-controlled RCT, the participants did not have chronic diarrhoea, heavy alcohol intake, use of diuretics and/or calcium antagonist drugs, previous oral magnesium supplementation, or ischaemic or renal diseases. Daily supplementation of 450 mg of magnesium reduced both SBP (−20.4 mmHg) and DBP (−8.7 mmHg), and the risk of bias in that trial was low for most of the evaluated domains (Table 2).

#### Vitamin C

**Figure 2:**
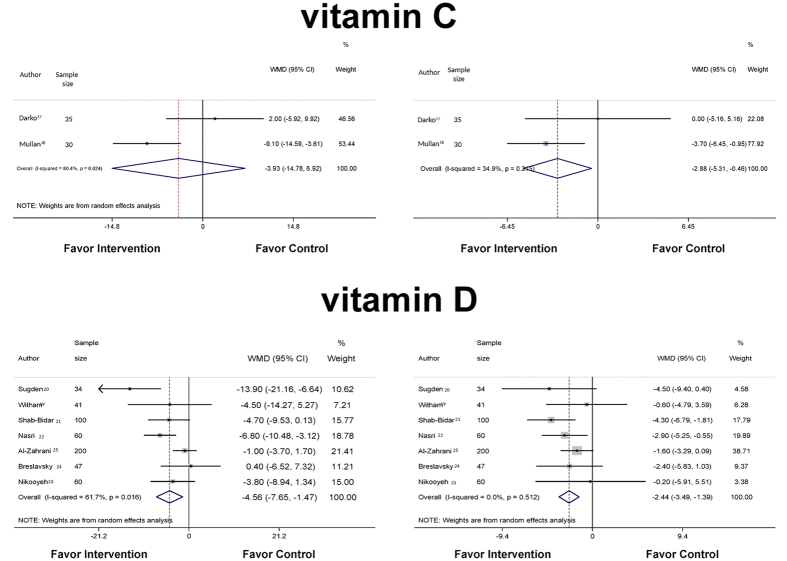
Forest plots of the effects of vitamin C and vitamin D on the blood pressure of patients with type 2 diabetes.

A total of 65 patients, 69% male, with ages ranging from 51.8 to 66.5 years old and with a mean BMI of 28.9 kg/m^2^, were evaluated in two studies^17^,^18^ examining the effects of vitamin C supplements (500 mg to 1,500 mg/day) on BP. When a meta-analysis was performed (Fig. 2), no effect of vitamin C on systolic BP was observed (WMD −3.93 mmHg; 95%CI −14.78, 6.92; *P* = 0.478). However, a reduction of −2.88 mmHg (WMD; 95%CI −5.31, −0.46; *P* = 0.020) in diastolic BP was observed compared with the control groups (Fig. 2). High heterogeneity in the systolic BP (I^2^ 80.4%; *P* = 0.024) but not in the diastolic BP (I^2^ 34.9%; *P* = 0.215) was observed between the studies.

#### Vitamin D

Seven studies (eight interventions)^19^,^20^,^21^,^22^,^23^,^24^,^25^ involving 542 patients with type 2 diabetes, 47% male, with ages ranging from 50.7 to 66.8 years, evaluated the effects of vitamin D on BP. Three of the studies^21^,^23^,^24^ did not include BMI values, and antihypertensive medication was described in only two of the RCTs^20^,^24^. Seven of the trials evaluated two different forms of vitamin D^19^,^20^,^21^,^22^,^23^,^24^,^25^. One study^20^ evaluated supplementation of ergocalciferol (vitamin D2), and six trials^19^,^21^,^22^,^23^,^24^,^25^ evaluated cholecalciferol (vitamin D3). In three of the interventions, the patients received a single dose of vitamin D2^20^ (100,000 IU) or vitamin D3^19^ (100,000 IU or 200,000 IU). One trial evaluated the effects of 50,000 IU/week of cholecalciferol for 12 weeks (a total dose of 600,000 IU)^22^, and the other study evaluated the effects of 225,000 IU of vitamin D3 (45,000 IU/week for 2 months and a single dose of 45,000 IU in the last month)^24^. One study^25^ evaluated the effects of daily supplementation with vitamin D3 by administering 1000 IU/day for 12 months, and two trials evaluated the effects of vitamin D3-fortified yogurt^21^,^23^ (500 IU D3 in 250 ml twice a day for 12 weeks; a total dose of 90,000 IU).

We pooled data from seven studies^19^,^20^,^21^,^22^,^23^,^24^,^25^ (Fig. 2), and the study by Witham *et al*.^19^ used the same control group for all tested dosages. Therefore, we included the data for only one vitamin D dosage (100,000 IU) to prevent the same individuals from being inappropriately included twice in the pooled estimate and because this dose was similar to doses used in other studies^20^,^21^,^23^. Our meta-analysis demonstrated that supplementation of vitamin D led to a 4.6 mmHg decrease (WMD; 95%CI −7.65, −1.47; P = 0.004; I^2^ 61.7%; *P* = 0.016) in systolic BP compared with the control group. The diastolic BP also decreased after intervention [WMD −2.44 mmHg (95%CI −3.49, −1.39; *P* < 0.001); I^2^ 0%; *P* = 0.512]. When the meta-analysis was performed using the data from the 200,000 IU arm instead of the 100,000 IU arm of the Witham *et al*. study^19^, the significance of the reductions in systolic BP (WMD −4.600 mmHg; 95%CI −7.714, −1.485, P = 0.004; I^2^ 61.8%, P = 0.015) and diastolic BP (WMD −2.398 mmHg; 95%CI −3.611, −1.186; P < 0.001; I^2^ 14.7%, P = 0.318) did not change.

Patients from the study by Shab-Bidar *et al*.^21^ along with patients from the study by Nasri *et al*.^22^ seemed to be younger than the patients from other studies. Additionally, the weekly vitamin dose used by Nasri *et al*.^22^ was higher than the doses used in the other studies, and the durations of follow-up of the studies were quite different (8 to 52 weeks). Therefore, our meta-regression analysis included vitamin D doses (90,000; 100,000; 225,000; 360,000; 600,000 IU) and age as covariates. Neither age, duration of follow-up nor doses were associated with changes in systolic BP (*P* = 0.482) or diastolic BP (*P* = 0.693). When we included the calculated daily dose of vitamin D instead of the total vitamin dose in the same model, there were also no associations between the doses and the changes in both systolic BP (*P* = 0.855) and diastolic BP (*P* = 0.684).

We performed two sensitivity analyses. In the first one, we excluded the study with the highest weighted mean difference [47.50% in SBP and 37.52% in DBP; ref. 21], and the significant reduction in BP remained for both systolic BP (WMD −7,081 mmHg; 95%CI −10.798, −3.365, *P* < 0.001; I^2^ 56.4%, *P* = 0.101) and diastolic BP (WMD −3.521 mmHg; 95%CI −5.482, −1.559, *P* = 0.002; I^2^ 16.5%, *P* = 0.302). In the second sensitivity analysis, the study with the most heterogeneity^25^ was excluded, and the significant reduction in BP also remained for both systolic BP (WMD −5,529 mmHg; 95%CI −8.679, −2.379, *P* < 0.001; I^2^ 44.2%, *P* = 0.110) and diastolic BP (WMD −2.965 mmHg; 95%CI −4.306, −1.624; *P* < 0.001; I^2^ 0.0%, *P* = 0.592).

The quality of the body of evidence in our systematic review, specifically for the studies included in the meta-analyses performed according to the GRADE approach, was considered moderate for vitamin D and low for vitamin C (Table 3).

## Discussion

In this systematic review of the effects of micronutrients on BP in patients with type 2 diabetes, we evaluated the effects of vitamin C, vitamin D, sodium, and magnesium. In pooled analyses, we demonstrated that supplemental vitamin C reduces diastolic BP and vitamin D reduces both systolic and diastolic BP.

### Vitamin C

A previous meta-analysis that evaluated 29 RCTs^31^ has already demonstrated a beneficial effect of supplemental vitamin C on BP. In that study, only five of the 29 included RCTs involved patients with diabetes, and in three of them, the supplemented vitamin C was combined with other micronutrients. In our meta-analysis, only patients with diabetes were included, and the individual effect of vitamin C on diastolic BP was demonstrated. However, the low quality of the included studies, especially the small number of studied patients, should be taken into in account. Therefore, we believe that the effects of vitamin C on BP in patients with type 2 diabetes remain unclear.

### Vitamin D

In the present meta-analysis of the effect of vitamin D on BP in patients with type 2 diabetes, we demonstrated a reduction in BP, especially in systolic BP. In all of the evaluated studies, the supplemented vitamin D was either ergocalciferol (vitamin D2) or cholecalciferol (vitamin D3). Although these vitamins are derived from different sources (plants, diet, or dermal synthesis), both require enzymatic conversion in the liver and kidney to active metabolites, and they were orally administered in all studies. Most importantly, they have the same expected biological effects^32^.

We identified three other meta-analyses of the effects of vitamin D on BP^33^,^34^,^35^. The first meta-analysis^33^ demonstrated a reduction in DBP of 3.1 mmHg in hypertensive patients. Of the 11 included RCTs, only one trial involved patients with diabetes. Moreover, the age range of the patients (48 to 74 years), the duration of the interventions (5 to 52 weeks), and the use of active (1–25 OHD) or inactive (vitamin D2, D3, UVB radiation) forms of vitamin D were associated with high heterogeneity of BP effects among the RCTs. Recently, Lee *et al*.^35^ have also demonstrated a small reduction in diastolic BP due to vitamin D supplementation in patients with type 2 diabetes. On the other hand, the meta-analyses of Beveridge *et al*.^34^ demonstrated that vitamin D did not reduce BP in the general population and in a subgroup analysis of 353 patients with diabetes. In contrast to our analysis, both of these meta-analyses^34^,^35^ included studies that examined the effects of paricalcitol, as well as intramuscular and parenteral vitamin D, on BP. Moreover, the results of studies in which BP was measured by office or ambulatory monitoring were evaluated together in the same meta-analysis^34^,^35^. Unlike our analysis, these meta-analyses^33^,^34^,^35^ did not include the studies of Nasri *et al*.^22^ and Al-Zahari *et al*.^25^.

In the current vitamin D meta-analysis, a moderate heterogeneity of effect was observed between RCTs. In addition, when we excluded the study^21^ that contributed the largest reduction in pooled BPs or the study with the greatest heterogeneity^25^, the results did not change. Moreover, it would be of interest to know if the effects of vitamin D on BP occur mainly in patients with hypovitaminosis. Most of the studies^19^,^20^,^21^,^22^,^23^,^24^,^25^ included patients who had serum concentrations of vitamin D that were lower than 50 nmol/l, and this cut-off value has been considered to be diagnostic of a vitamin D deficiency^36^. However, we cannot make generalizations concerning the observed beneficial effects of vitamin D on BP in patients with sufficient levels of vitamin D. Although most of the studies included in our meta-analysis had a low risk of bias, the quality of the body of evidence in the current vitamin D meta-analysis was classified as moderate^29^. This means that additional studies are still desirable to confirm our results.

Observational and experimental data favour the concept that vitamin D is associated with the pathogenesis of arterial hypertension^37^,^38^. A possible mechanism for this link involves the inhibition of the rennin-angiotensin-aldosterone system by vitamin D. Additionally, in the presence of hypovitaminosis D, an alternative mechanism could be related to the secondary hyperparathyroidism and relative hypocalcemia that are commonly seen in these patients^38^.

### Sodium

The effects of the reduction of sodium intake on BP, particularly in patients with diabetes, were described in only one study published at the end of the 1980s^15^. Although salt restriction in hypertensive patients with or without diabetes is highly recommended^6^,^9^,^11^, the salt restriction phase of that study was short. Moreover, that trial involved a small sample of patients. Therefore, the specific effects of sodium restriction on BP in type 2 diabetic patients could not be properly evaluated.

### Magnesium

Concerning magnesium supplementation, only one study described a reduction in BP in diabetic patients^16^. The absence of side effects of magnesium supplementation in this trial (e.g., nausea, vomiting, hypocalcaemia, or even cardiovascular effects) was probably due to the presence of hypomagnesaemia and the use of a magnesium dose that was similar to the recommendations for daily intake^32^. Even though that study was well conducted with almost no bias, we believe that their results are not applicable to type 2 diabetic patients who do not have a magnesium deficiency.

### Limitations and strengths of our study

One limitation of our systematic reviews and meta-analyses was the inclusion of few reports, despite the large number of revised trials. However, because the evaluated data may comprise the currently available literature on this topic, we believe that our study provides important information that adds to the current knowledge of the impact of micronutrients on BP in patients with diabetes. Another possible limitation is that the BPs were evaluated only in the office in all of the included RCTs. Office BP measurements comprise the usual assessment in clinical practice, but a more comprehensive evaluation of BP homeostasis could be obtained by continuous BP monitoring^38^. Finally, we could not find any long-term RCTs of the effects of micronutrients on BP in patients with diabetes, as well as analyses of hard outcomes such as mortality.

The present systematic review and meta-analyses were conducted in accordance with the Cochrane^26^,^28^ and PRISMA^27^ guidelines. A very extensive literature search strategy was used, regardless of language, that included both micronutrient and macronutrient terms. In addition, we excluded studies that evaluated more than one micronutrient intervention together or any combined nutritional interventions in order to assess the actual effect of the micronutrient on BP.

## Conclusion

In conclusion, our systematic review and meta-analyses of RCTs demonstrated that the supplementation of vitamin D may reduce both systolic and diastolic BP in patients with type 2 diabetes. The effect of vitamin C, although significant, was not definitively demonstrated. Finally, our data suggest that these interventions could be adopted to treat high BP in patients with diabetes, especially in the presence of hypovitaminosis D. However, further studies with long-term follow-up periods and large samples of hypertensive type 2 diabetic patients are needed.

## Additional Information

**How to cite this article:** de Paula, T. P. *et al*. Effect of individual micronutrients on blood pressure in patients with type 2 diabetes: systematic review and meta-analysis of randomized clinical trials. *Sci. Rep.* **7**, 40751; doi: 10.1038/srep40751 (2017).

**Publisher's note:** Springer Nature remains neutral with regard to jurisdictional claims in published maps and institutional affiliations.

## Supplementary Material

Supplementary Information

## Figures and Tables

**Table 1:** Characteristics of the included studies of the effects of micronutrients on blood pressure in patients with type 2 diabetes.

Author Year	Design/Trial Duration		Diabetes duration (years)	BMI (kg/m^2^)	Baseline BP (mmHg)	Diet characteristics and micronutrient measurements	BP change (mean, mmHg)	Medications in use (% of users)
Sodium								
Dodson 1989	Parallel/3 months (1^st^ phase of study)	n = 34	4.6 ± 4.3	NA	SBP	Intervention	Intervention	Oral hypoglycaemic: 18%
		67.6% males			I.179.7 ± 18.2	Advised to restrict daily dietary sodium intake	SBP: −19.2 ± 13.5	Atenolol: 12%
		I.61.9 ± 7.5 y			C.173.8 ± 20.3	Sodium intake* (g/NaCl)	DBP: −3.8 ± 6.9	
		C.61.1 ± 6.3 y				Baseline: 11.7 g ± 3.9		
		Hypertensive subjects				End-of-study: 8.04 ± 2.2		
						Change: −3.44 ± 2.48		
					DBP	Control	Control	
					I.91.4 ± 11.1	Usual diet for diabetes (ADA)	SBP: −6.2 ± 13.1	
					C.92.4 ± 10.9	Sodium intake* (g/NaCl)	DBP: −2.0 ± 7.2	
						Baseline: 10.8 g ± 1.6		
						End-of-study: 10.6 ± 1.9		
						Change: −0.2 ± 1.13		
Magnesium								
Guerrero Romero 2009	Parallel/3 weeks	n = 82	8.6 ± 0.9	29.1 ± 1.3	SBP	Intervention	Intervention	Glibenclamide: 100%
		48.1% males			I.161 ± 26	Daily oral supplement: 2.5 MgCl (=0.45 g Mg)	SBP: −20.4 ± 15.9	
		I.58.9 ± 9.0 y			C.154.5 ± 21.2	Magnesium nmol/l^†^	DBP: −8.7 ± 16.3	
		C.60.5 ± 9.4 y				Baseline: 0.62 ± 0.10		
		Hypertensive subjects with low serum magnesium and without use of diuretics				End-of-study: 0.81 ± 0.11		
						Change: 0.18 ± 0.10		
					DBP	Control/nmol/l^†^	Control	
					I.88.4 ± 14.5	Baseline: 0.61 ± 0.10	SBP: −4.7 ± 12.7	
					C.84.9 ± 12.4	End-of-study: 0.68 ± 0.11	DBP: −1.2 ± 12.6	
						Change: 0.08 ± 0.14		
Vitamin C								
Darko 2002	Parallel/3 weeks	n = 35	I.9.3 ± 1.2	29.1 ± 1.3	SBP	Intervention	Intervention	Diuretics = 9%
		66% males	C.7.8 ± 0.6		I.141.0 ± 5.0	Daily oral supplement: 1.5 g ascorbic acid	SBP 0.0 ± 13.4	ACE inhibitors = 11%
		I.56.6 ± 1.1 y			C.138.0 ± 4.0	Plasma ascorbate/μmmol	DBP1.0 ± 8.0	Sulphonylureas = 23%
		C.55.5 ± 1.8 y				Baseline: 58 ± 6		Metformin = 37%
						End-of-study: 122 ± 10		
						Change: 64 ± 6.5		
					DBP	Control (placebo)	Control	
					I.80.0 ± 2.0	Plasma ascorbate/μmmol	SBP: −2.0 ± 10.3	
					C.76.0 ± 3.0	Baseline: 51 ± 5	DBP: 1.0 ± 7.6	
						End-of-study: 53 ± 5		
						Change: 2 ± 3		
Mullan 2002	Parallel Double Blind/4 weeks	n = 30	NA	28.6 ± 4.3	SBP	Intervention	Intervention	NA
		73% males			I.130.1 ± 12.4	Daily oral supplement: 500 mg ascorbic acid	SBP: −10.1 ± 7.9	
		I.57.9 ± 6.6 y			C.129.7 ± 11.7	Plasma ascorbic acid/μmmol/l	DBP: −4.4 ± 3.7	
		C.61.0 ± 6.5 y				Baseline: 43.3 ± 19.3		
						End-of-study: 78.1 ± 19.5		
						Change: 34.8 ± 12		
					DBP	Control (placebo)	Control:	
					I.80.5 ± 6.2	Plasma ascorbic acid: NA	SBP: −1.0 ± 7.4	
					C.85.1 ± 6.4		DBP: −0.6 ± 3.9	
Vitamin D								
Sugden 2008	Parallel double blind/8 weeks	n = 34	NA	31.7 ± 5.4	SBP	Intervention	Intervention	ACE inhibitor or angiotensin blocker = 62%
		53% males			I.145 ± 9.2	Single dose supplement: 100,000 IU D2 (*12*,*500 IU*/*week*)	SBP: −7.3 ± 11.8	Metformin = 53%
		64.2 ± 9.9 years old plasma vitamin D < 50 mmol/l			C.137 ± 14.1	Serum 25 OHD/nmol/l	DBP: −2.2 ± 8.6	Insulin = 18%
						Baseline: 40.2 ± 10.3		
						Change: 22.9 ± 16.6		
					DBP	Control	Control	
					I.82 ± 10.5	Single dose placebo Miglyol^®^ oil	SBP: 6.6 ± 9.7	
					C.79 ± 6.0	Serum 25OHD/nmol/l	DPB: 2.3 ± 5.7	
						Baseline: 36.4 ± 8.5		
						Change: 7.6 ± 12.5		
Witham 2010	Parallel/16 Weeks	n = 61	NA	I.31.1 ± 6.7	SBP	Intervention: 1	Intervention - 1	NA
		68% males		C.33.3 ± 7.1	I.149.6 ± 24.8	Single dose supplement: 100,000 IU D3 (*6*,*250 IU*/*week*)	SBP: −8.2 ± 15.2	
		I.65.3 ± 9.7 y			C.143.9 ± 24.4		DBP: −3.6 ± 8.6	
		C.66.7 ± 9.7 y			DBP	Serum 25 OHD/nmol/l		
					I.80.7 ± 14.3	Baseline: 41 ± 14		
					C.80.3 ± 9.7	End-of-study: 63 ± 20		
						Change: 23.0 ± 18.4		
					SBP	Intervention: 2	Intervention - 2	
					I.145.1 ± 25.0	Single dose supplement: 200,000 IU D3 (*12*,*500 IU*/*week*)	SBP: −5.6 ± 15.7	
					C.143.9 ± 24.4		DBP: −3.1 ± 8.6	
					DBP	Serum 25 OHD/nmol/l		
					I.80.7 ± 14.3	Baseline: 48 ± 21		
					C.80.3 ± 9.7	End-of-study: 79 ± 31		
						Change: 31.0 ± 19.0		
						Control (for interventions 1 and 2)	Control	
						Single dose placebo Miglyol^®^ oil	SBP: 2.5 ± 14.6	
						Serum 25OHD (nmol/l)	DBP: −1.4 ± 6.0	
						Baseline: 45 ± 17		
						End-of-study: 54 ± 20		
						Change: 9 ± 12		
ShabBidar 2011	Parallel double blind/12 weeks	n = 100	I.8.3 ± 4.6	NA	SBD	Intervention	Intervention	Oral antihyperglycemic = 100% (metformin, glibenclamide, glitazones)
		43% males	C.7.0 ± 5.2		I.125.7 ± 14.4	Vitamin D3-fortified yogurt: 170 mg calcium and 500 IU D3/250 ml, twice/day (total dose 90,000 IU)	SBP: −7.2 ± 12.8	
		I.52.4 ± 8.4 y			C.128.2 ± 16.6		DBP: −5.1 ± 6.2	
		C.52.6 ± 6.3 y			DBP	Serum 25OHD/nmol/l		
					I.78.5 ± 10.3	Baseline: 38.5 ± 20.2		
					C.77.8 ± 10.8	End-of-study: 72 ± 23.5		
						Change: 33.5 ± 14.2		
						Control	Control	
						Plain yogurt: 170 mg calcium without vitamin D3/250ml	SBP: −2.5 ± 11.8	
						Serum 25OHD/nmol/l	DBP: −0.8 ± 6.5	
						Baseline: 38.5 ± 22.8		
						End-of-study: 33.4 ± 22.8		
						Change: −4.6 ± 14.4		
Nikooyeh 2011	Parallel 12 weeks	n = 90	NA	I.29.9 ± 4.7	SBP	Intervention (*n* = *30*)	Intervention	Oral antihyperglycemic = 100% (metformin, glibenclamide, glitazones)
		39% males		C.29.2 ± 4.4	I.131.5 ± 21.6	Vitamin D3-fortified yogurt: 150 mg calcium and 500 IU D3/250 ml, twice/day (total dose 90,000 IU) (*7*,*500 IU*/*week*)	SBP: −3.4 ± 10.3	
		50.7 ± 6.1 years old			C.127.3 ± 14.8		DBP: 0.3 ± 14.0	
					DBP	Serum 25OHD/nmol/l		
					I.77.5 ± 20.0	Baseline: 44.4 ± 28.7		
					C.77.5 ± 10.6	End-of-study: 77.7 ± 28.6		
						Change: 33.3 ± 18.1		
						Control (n = 30)	Control	
						Plain yogurt: 150 mg calcium without vitamin D3/250 ml	SBP: 0.4 ± 10.0	
						Serum 25OHD/nmol/l	DBP: 0.5 ± 7.7	
						Baseline: 41.6 ± 44.5		
						End-of-study: 37.2 ± 44.0		
						Change: −4.4 ± 28.0		
Breslavsky 2013	Parallel placebo/controlled 52 weeks	n = 47	NA	NA	SBP	Intervention	Intervention	Metformin = 49%
		46.8% males			I.154.2 ± 21.5	Daily supplement of vitamin D3 1000 mg (total dose 360,000 IU) (*6*,*667 IU*/*week*)	SBP: −10.7 ± 12.8	Sulfonylurea = 23,4%
		I.66.8 ± 9.2 y			C.151.8 ± 18.0		DBP: 0.1 ± 5.3	
		C.65.8 ± 9.7 y			DBP	Serum 25OHD/ng/ml		
					I.76.2 ± 8.8	Baseline: 11.8 ± 10.9		
					C.72.2 ± 10.8	End-of-study: 17.6 ± 11.5		
						Change: 5.8 ± 7.1		
						Control Placebo (microcrystalline cellulose)	Control	Diuretics = 34%
						Serum 25OHD/ng/ml	SBP: −11.1 ± 11.4	ACE inhibitors = 66%
						Baseline: 11.7 ± 6.5	DBP: 2.5 ± 6.6	B-Blockers = 51%
						End-of-study: 14.0 ± 5.9		
						Change: 2.3 ± 3.9		
Al-Zahari 2013	Parallel placebo/controlled 12 weeks	n = 200	NA	I.31.3 ± 4.6	SBP	Intervention	Intervention	NA
		45% males		C.32.0 ± 5.7	I.123.4 ± 15.8	45.000 IU of vitamin D3 every week for 2 months and a single dose of 45000 IU in the last month (total dose 225,000 IU) (*18*,*750 IU*/*week*)	SBP: −1 ± 9.8	
		I.56.9 ± 9.4 y			C.124.0 ± 15.4		DBP: −3.2 ± 6.6	
		C.52.5 ± 8.1 y			DBP	Serum 25OHD/nmol/l		
					I.76.4 ± 10.8	Baseline: 25.3 ± 15.8		
					C.75.3 ± 9.2	End-of-study: 82.8 ± 31.7		
						Change: 57.5 ± 21.3		
						Control	Control	
						Placebo (microcrystalline cellulose)	SBP: 0 ± 9.6	
						Serum 25OHD/nmol/l	DBP: −1.6 ± 5.5	
						Baseline: 22.0 ± 15.2		
						End-of-study: 55.0 ± 37.5		
						Change: 33 ± 27.2		
Nasri 2014	Parallel double blind placebo controlled/12 weeks	n = 60	NA	NA	SBP	Intervention	Intervention	NA
		28.3% males			I.121.0 ± 13.0	50,000 IU of vitamin D3 per week (*total dose 600*,*000 IU*)	SBP: −11 ± 7.9	
		55 ± 10.7 years old			C.118.8 ± 11.0		DBP: −4.2 ± 4.8	
					DBP	Serum 25OHD/nmol/l		
					I.80.5 ± 8.0	Baseline: 83.9 ± 52		
					C.80.3 ± 7.0	End-of-study: 164 ± 57		
						Change: 80.1 ± 34.8		
						Control	Control	
						Serum 25OHD/nmol/l	SBP: −4.2 ± 6.6	
						Baseline: 105.7 ± 64	DBP: −1.3 ± 4.5	
						End-of-study: 115.8 ± 94		
						Change: 10.1 ± 57.5		

Abbreviations: ADA = American Diabetes Association; BP = blood pressure; C = control group; DBP = diastolic blood pressure; DM = diabetes mellitus; I = intervention group; NA = not available; SBP = systolic blood pressure; y = years old; *NaCl intake estimated by 24-h urinary sodium (g), ^†^magnesium intake based on 24-h urinary magnesium (mmol/L).

Data are expressed as the mean (standard deviation).

**Table 2:** Assessment of the quality of the studies included in the systematic review: a summary of risk of bias (Cochrane Handbook for Systematic Reviews of Interventions Version 5.1.0).

	Selection Bias		Performance Bias	Detection Bias	Attrition Bias	Reporting Bias	Other Bias
	Random sequence generation	Allocation concealment	Blinding of participant and personnel	Blinding of outcome assessment	Incomplete outcome data	Selective reporting	Diet/supplement compliance assessment
Sodium							
Dodson^15^	unclear	unclear	low*	low	low	low	low
Magnesium							
Guerrero-Romero^16^	low	unclear	low	unclear	low	low	low
Vitamin C							
Darko^17^	unclear	unclear	low	unclear	low	low	low
Mullan^18^	unclear	unclear	low	unclear	low	low	low
Vitamin D							
Witham^19^	low	low	low	unclear	low	low	low
Sugden^20^	low	low	low	unclear	low	low	low
Shab-Bidar^21^	unclear	unclear	low	unclear	low	low	low
Nasri^22^	low	low	low	unclear	low	low	low
Al-Zahrani	low	low	high	unclear	high	unclear	unclear
Nikooeh	unclear	unclear	unclear	unclear	unclear	unclear	low
Breslavsky	unclear	unclear	low	unclear	unclear	low	unclear

*Blinding of participants and personnel was not applicable because the intervention was dietary advice only.

**Table 3:** GRADE evidence profiles for the meta-analysis of the effects of vitamin C and vitamin D on blood pressure in patients with type 2 diabetes.

Vitamin C										
Quality assessment							Summary of Findings			
Participants (studies) Follow-up	Risk of bias	Inconsistency	Indirectness	Imprecision	Publication bias	Overall quality of evidence	Study event rates (%)		Relative effect (95% CI)	Anticipated absolute effects
							Intervention	Control		
Systolic Blood Pressure (IMPORTANT OUTCOME; Better indicated by lower values)										
65 (2 studies) 3.5 weeks	Serious^1^	Serious^2^	not serious	Serious^3^	undetected	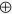 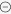 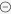 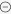 **VERY LOW** due to risk of bias, inconsistency, imprecision	32	33	—	The mean systolic blood pressure in the intervention group was **3.93** lower (14.78 **lower** to 6.92 higher)
Diastolic Blood Pressure (IMPORTANT OUTCOME; Better indicated by lower values)										
65 (2 studies) 6.35 weeks	serious^1^	serious^2^	no serious indirectness	no serious imprecision	undetected	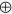 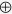 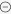 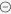 **LOW**^1^,^2^ due to risk of bias, inconsistency	32	33	—	The mean diastolic blood pressure in the intervention group was **2.88 lower** (2.88 to 0.46 lower)
**Vitamin D**										
Systolic Blood Pressure (Better indicated by lower values)										
542 (7 studies) 3–52 weeks	no serious risk of bias	Serious^2^	no serious indirectness	no serious imprecision	undetected	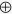 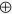 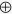 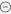 **MODERATE** due to inconsistency	272	270	—	The mean systolic blood pressure in the intervention group was **4.558 lower** (7.65 to 1.465 lower)
Diastolic Blood Pressure (IMPORTANT OUTCOME; Better indicated by lower values)										
542 (7 studies) 3–52 weeks	no serious risk of bias	no serious inconsistency	no serious indirectness	no serious imprecision	undetected	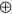 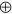 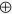 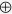 **HIGH**	272	270	—	The mean diastolic blood pressure in the intervention group was **2.437 lower** (3.487 to 1.387 lower)

^1^Most of the information concerning randomization and blinding was unclear in the analyses of the individual trials.

^2^High heterogeneity.

^3^Few patients analysed.
