# Supplementary material for: Effects of individual micronutrients on blood pressure in patients with type 2 diabetes: a systematic review and meta-analysis of randomized clinical trials
Source: Sci Rep. 2017 Jan 13;7:40751. doi: 10.1038/srep40751 (PMC5233957; doi:10.1038/srep40751)
Supplement: Supplementary Information [file srep40751-s1.pdf]

Effect of individual micronutrients on blood pressure in type 2 diabetes: systematic review and meta-analysis of randomized clinical trials.

Authors: Tatiana P. de Paula, RD, PhD[1], Caroline K. Kramer, MD, PhD[2], Luciana V. Viana, MD, PhD[1], Mirela J. Azevedo, MD, PhD[1]

SUPPLEMENTARY INFORMATION

("Micronutrients"[Mesh]) OR ("Phosphorus"[Mesh] OR "Phosphorus, Dietary"[Mesh])) OR ("Sodium"[Mesh] OR "Sodium, Dietary"[Mesh])) OR "Chromium"[Mesh]) OR "Calcium"[Mesh]) OR ("Iron"[Mesh] OR "Iron, Dietary"[Mesh])) OR ("Magnesium"[Mesh] OR "Magnesium Chloride"[Mesh])) OR ("Potassium"[Mesh] OR "Potassium, Dietary"[Mesh])) OR ("Vitamins"[Mesh] OR "Vitamin B Complex"[Mesh])) OR "Vitamin A"[Mesh]) OR "Ascorbic Acid"[Mesh]) OR "Vitamin B 6"[Mesh]) OR "Vitamin B 12"[Mesh]) OR "Vitamin D"[Mesh]) OR "Vitamin E"[Mesh]) OR "Zinc"[Mesh]) OR "Carbohydrates"[Mesh]) OR "Proteins"[Mesh]) OR "Lipids"[Mesh]) OR "Dietary Fiber"[Mesh]) OR "Ethanol"[Mesh]) OR "Alcohols"[Mesh]) OR "Alcohol Drinking"[Mesh]) OR ("Cholesterol"[Mesh] OR "Cholesterol, Dietary"[Mesh])) OR Fish oil OR Fatty acids OR omega-3 OR omega 6 OR Tea OR cocoa OR chocolate AND "Blood Pressure"[Mesh]) OR "Hypertension"[Mesh]) AND "Diabetes Mellitus"[Mesh].

Online-only Supplementary Medline search strategy:

Vitamin C: systolic and diastolic BP

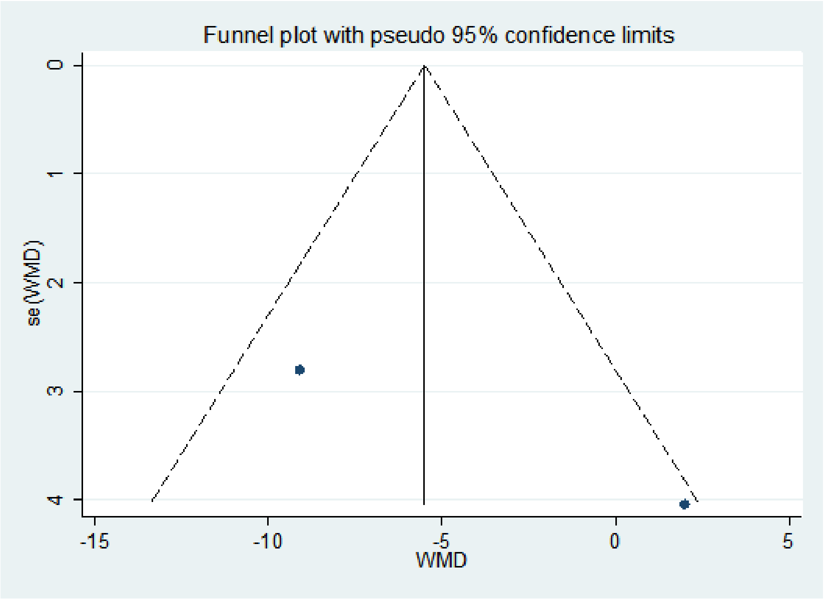

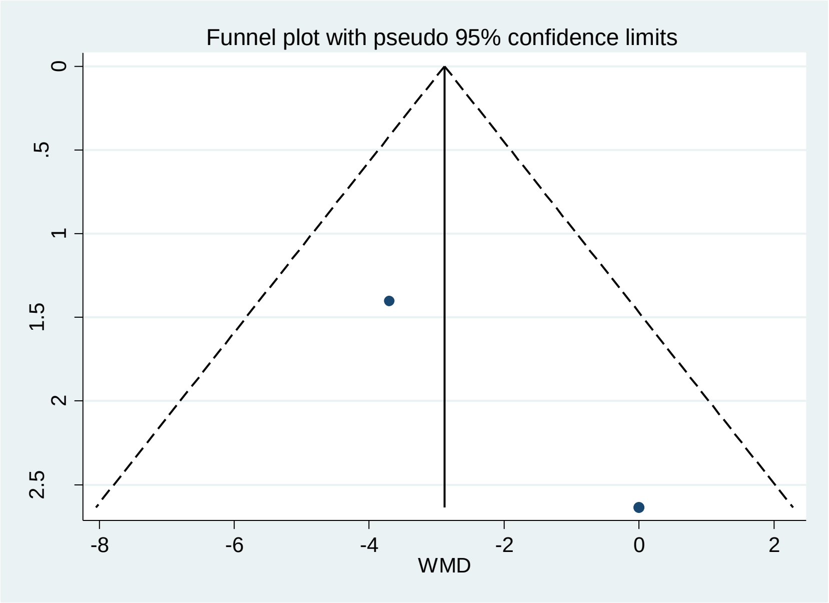

B) Vitamin D: systolic and diastolic BP

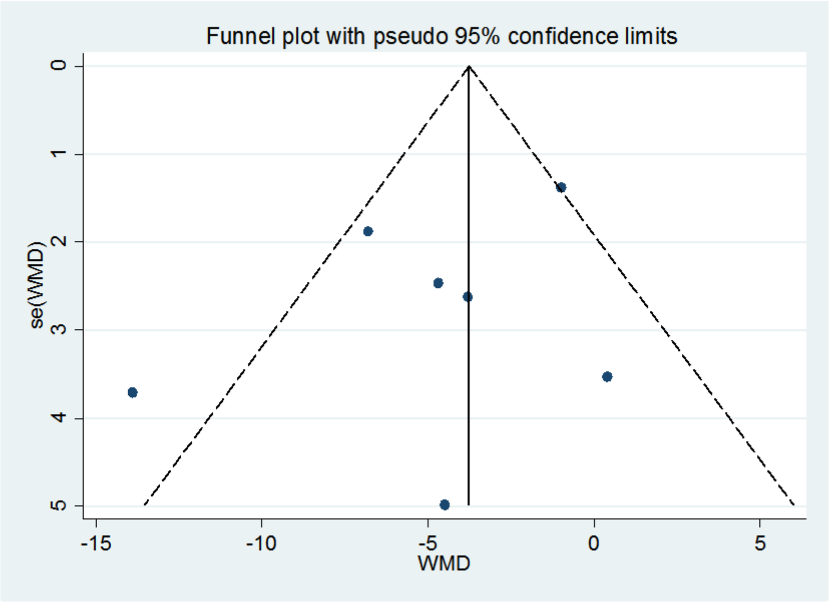

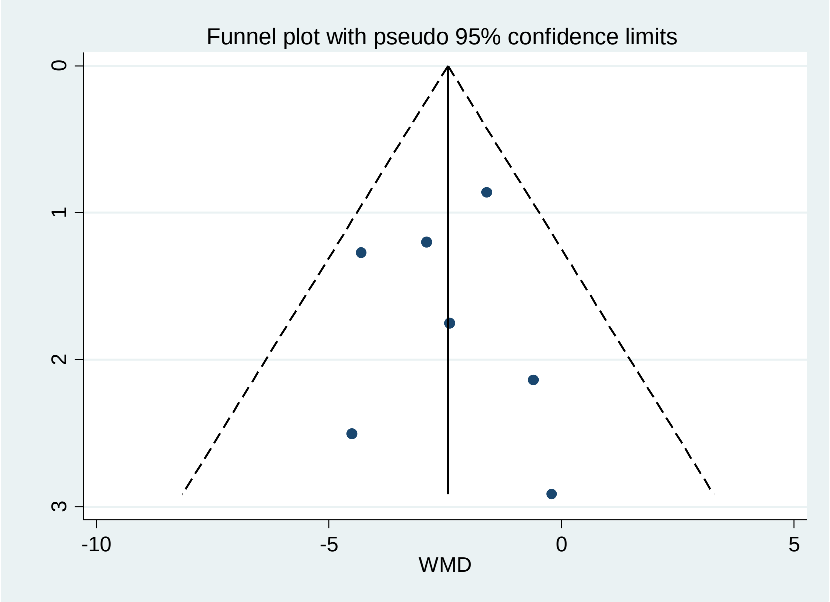

Funnel plots diagram of publication bias of the meta-analyses of the effect of individual micronutrients on blood pressure in type 2 diabetes: systematic review and meta-analysis of randomized clinical trials. A) vitamin C and B) vitamin D.

BP = blood pressure
